# Postoperative Visual Loss Following Spine Surgery: A Case Report

**DOI:** 10.31729/jnma.4539

**Published:** 2019-08-31

**Authors:** Sabin Bhandari, Krishna Pokharel, Birendra Prasad Sah

**Affiliations:** 1 Department of Anaesthesiology and Critical Care, B.P. Koirala Institute of Health Sciences, Dharan, Nepal

**Keywords:** *ischaemic optic neuropathy*, *postoperative visual loss*, *spine surgery*

## Abstract

Postoperative visual loss is a rare but devastating complication of non-ophthalmic surgery. Its aetiology is poorly understood and multiple associated factors have been proposed. We present a report of a 33-year-old female who developed irreversible diminution of vision on the right eye (non-arteritic-posterior-ischemic-optic-neuropathy) following general anaesthesia for pedicle screw fixation and plating for fracture vertebrae and hip in prone position and then screw placement for fracture calcaneum in supine position. The vision loss, limited to finger count close to face on the right eye, did not improve till follow-up at one-year. The combination of mild intraoperative hypotension, anaemia, prone positioning, prolonged surgery and anaesthesia may have contributed to postoperative visual loss in our patient.

## INTRODUCTION

Postoperative visual loss (POVL) is a rare, devastating complication of non-ophthalmic surgery. Its incidence is variable from 0.05 to 1.3% and mostly reported after cardiac (incidence 0.09%) and spinal (incidence 0.03%) surgery.^1,2^ However, the exact incidence and aetiology remains unknown as most cases of postoperative vision loss following spine surgery are mentioned as case reports, which are difficult to obtain as the cases are often subject to malpractice claims, resulting in a lack of public access to case reports.

## CASE REPORT

A 33-year-old female, weighing 70 kg, was scheduled for pedicle screw fixation at T11-L3, open reduction internal fixation for fracture hip and closed reduction and cortico-cancellous screw for calcaneum fracture for a five-day-old traumatic anterior wedge compression fracture L1 vertebrae without neurological deficit, fracture left iliac blade and fracture calcaneum without distal neurovascular damage.

Her pre-anaesthetic check-up revealed anaemia (hemoglobin 9.4 gm/dl). After induction of general anaesthesia, her eyes were padded with cotton gauze and she was placed prone. The head was placed on a horse shoe in a neutral position. We ensured that pressure was not exerted on the eyes. The abdomen was also checked for free movement. After three and half hours of prone positioning, she was made supine for surgery on the hip and calcaneum. We followed the routine departmental anaesthesia protocol for induction, maintenance and reversal of anaesthesia. We maintained mean arterial pressure (MAP) from 60 to 70 mmHg (20% below baseline) during the manipulation of spine with IV esmolol 5 mg boluses and IV nitroglycerin 50 -100 mcg boluses for two hours. MAP was maintained close to baseline for the remaining period. Heart rate was maintained at 100-120/min. Estimated blood loss was 700 ml. One unit of packed red cells was transfused after the hemostasis was secured. Urine output was 75–100 ml/h. The surgery lasted for four and half hours.

In the orthopaedics in-patient unit, the patient complained of painless diminution of vision in the right eye. An ophthalmologist was consulted. In the right eye, vision was limited to finger count close to face. Intraocular pressure was normal. A dense apparent pupillary defect was present. Anterior segments were normal. Fundal examination showed normal discs and maculae without swelling or pallor or retinal hemorrhage. Visual evoked potential showed perception of light with absent visual evoked response. There was no abnormality on the left eye. A diagnosis of non-arteritic posterior ischemic optic neuropathy of right eye was made. IV methylprednisolone 250 mg four times a day for three days followed by tapering dose of tab prednisolone over 15 days was administered. Other aspects of recovery from surgery was unremarkable. On regular follow-up for one year in the ophthalmology out-patient-service, her visual acuity did not improve.

## DISCUSSION

The aetiology of post-operative visual loss is poorly understood. Proposed risk factors include obesity, cardiovascular disease, hyper-lipidemia, diabetes, smoking, male gender, intraoperative hypotension, prone positioning, less usage of colloids, prolonged procedures and anaesthesia.^3^

The American Society of Anaesthesiologists (ASA) closed claim database analysis of 93 patients with postoperative visual loss after spine surgery from 1999 to June 2005 revealed 89% had ischaemic optic neuropathy (ION) while 10% had central retinal arterial occlusion. Among the patients developing ION, majority (60%) had posterior ischaemic optic neuropathy (PION).^4^

Our patient was anaemic. We also induced deliberate hypotension (MAP 20 % below baseline but around 60 – 70 mm Hg) for two hours. Although these two are the most commonly blamed risk factors, the ASA report does not suggest any independent effect of anaemia or intraoperative mean arterial pressure drop of more than 40% below baseline for 30 congruent or additive minutes on postoperative blindness.^5^ Our patient did not have decreased urine output or ECG changes that may reflect reduced end organ perfusion due to hypotension.

Another risk factor for postoperative visual loss is increased intraocular tension by direct pressure on the globe or raised central venous pressure (CVP) due to head-down position, rotation of head or direct pressure on the abdomen during prone positioning. We had ensured that these factors were absent. However, the prone position itself can significantly increase intraocular pressure which could be normalized by 10° reverse trendelenberg position.^6,7^ We did not take this precaution. An analysis of the reports of spine procedures obtained from the Anaesthesia Closed Claims Project database (2000 to 2014) found that prone positioning and surgical duration of ≥4 hours were associated with permanent disabling injuries to nerves, spinal cord, and eyes or visual pathways.^8^ The duration of surgery in our patient was four and a half hours.

It is likely that the combination of preoperative anaemia, intraoperative mild to moderate hypotension, prone positioning, prolonged surgery and anaesthesia resulted in a reduced perfusion pressure to the optic nerve and hence, visual loss in our patient. We strongly feel that we should have corrected the preoperative anaemia and also staged the last part of surgery after a few days instead of performing it in a single setting. The practice of deliberate hypotension for all spine surgery in prone position also warrants some caution.

In conclusion, both anaesthesiologist and surgeon need to be aware that a number of risk factors which, when combined, can cumulatively increase the risk of postoperative visual loss. A thorough preoperative evaluation to identify high risk patients should be performed and they should be informed about the risk of POVL. Whenever possible, staged procedure to reduce surgical duration should be considered. During surgery, a 10° reverse trendelenburg position should be maintained with the neck in a neutral forward position. Direct ocular pressure must be prevented, and the anaesthesiologist should have unobstructed access to the patient's eyes for repeat positioning checks. Normocarbia should be maintained, prolonged decrease in blood pressure should be corrected and deliberate hypotension should only be used with extreme caution at high risk patients. Monitoring the haemoglobin values frequently and avoiding anaemia are other preventive measures. Postoperatively, the vision of a high-risk patient should be assessed once the patient becomes alert and if there is concern regarding potential visual loss, an urgent ophthalmologic consultation should be obtained. Also, maintaining 30–45° head-up position postoperatively, if feasible, to limit orbital oedema is another preventive measure.1,2,6,7,8,9,10

## Consent:

**JNMA Case Report Consent Form**was signed by the patient and the original is attached with the patient's chart.

## Conflict of Interest:

**None.**
